# A qualitative descriptive study of providers’ perspectives on human papillomavirus vaccine administration among Latino/a adolescents in South Texas clinics: barriers and facilitators

**DOI:** 10.1186/s12889-022-12837-2

**Published:** 2022-03-05

**Authors:** Daisy Y. Morales-Campos, Bertha E. Flores, Erin Donovan, Suzanne Burdick, Deborah Parra-Medina, Jessica A. Kahn

**Affiliations:** 1 Latino Research Institute, The University of Texas at Austin, 210 W. 24th Street, GWB 1.102C, F9200, Austin, TX 78712 USA; 2 School of Nursing, UT Health San Antonio, San Antonio, USA; 3 Department of Communication Studies, The University of Texas at Austin, Austin, USA; 4 Cincinnati Children’s Hospital Medical Center and the Department of Pediatrics, University of Cincinnati College of Medicine, Cincinnati, USA

**Keywords:** Cancer prevention, Healthcare providers, HPV vaccination, Latino adolescents, Qualitative description

## Abstract

**Background:**

South Texas Latinas experience higher cervical cancer incidence and mortality compared to Latinas nationwide. Despite the availability of effective human papillomavirus vaccines, South Texas Latino/a adolescents sub-optimally complete the series. Research shows provider recommendation strongly predicts vaccine uptake, but minority adolescents are less likely to report that their provider recommended the vaccine and series completion. There is also scant information on the HPV vaccine administration process in clinic practices providing vaccination services to Latino adolescents with limited access to healthcare resources. The purpose of the study was to describe providers’ experience with administering the HPV vaccine to Latino/a patients in their practices.

**Methods:**

The study used qualitative description to describe the experience of 15 South Texas healthcare providers (doctors and nurses) with the process of HPV vaccine administration in their practices. We conducted open ended, audio-recorded interviews, which were subsequently transcribed verbatim and uploaded into Atlas.(ti) 7.0 for analysis. The interviews yielded detailed descriptions of barriers and facilitators that could potentially impact HPV vaccine uptake.

**Results:**

Providers identified parental exposure to provider recommendation as enhancing HPV acceptance and existing policies and implementation of evidence-based practices as facilitators of HPV vaccine uptake. Barriers ranged from parental fears of adolescent sexual activity and potential vaccine side effects to lack of transportation and the cost of the vaccine.

**Conclusion:**

These findings reflect barriers and facilitators to administering the HPV vaccine previously identified and also highlight issues unique to the situation among Latinos in South Texas. Implications include the need to design and implement efforts to improve provider-parent communication and enhance parental and adolescent patients’ understanding of and confidence in the HPV vaccine. Furthermore, policy changes are needed to rectify organizational/structural challenges to HPV vaccine administration.

## Background

Despite the availability of highly effective human papilloma virus (HPV) vaccines, in the United States (U.S.) there are an estimated 79 million individuals currently infected with HPV, which is responsible for approximately 91% of cervical cancer cases in women [1–3]. Of note, there are significant racial, ethnic and socioeconomic disparities in cervical cancer incidence and mortality, and these disparities also vary by geographic region [4]. Latinas in Texas, compared to those nationwide, experience higher cervical cancer incidence (11 vs. 9/100,000) and mortality (3 vs. 2/100,000) [5–7]. Vaccinating adolescents aged 11–17 against HPV is known as an evidence-based intervention that reduces mortality [8, 9] and has led the Advisory Committee on Immunization Practices (ACIP) to recommend HPV vaccines for male and female adolescents (ages 11–17) [10]. Despite these recommendations and widespread availability of HPV vaccines [11], vaccine uptake is suboptimal [12, 13].

In the U.S., the Centers for Disease Control and Prevention (CDC) release a recommended immunization schedule, which is developed by a panel of 15 medical and public health experts known as the ACIP. Each state takes the schedule and mandates its own list of vaccines required for school entry [14]. The U.S. federal government funds the Vaccines for Children (VFC) program to provide free vaccines for U.S. children who are uninsured, underinsured (insurance does not cover immunization), on Medicaid, or who are American Indians/Alaska Natives [15]. The CDC uses VFC funds to purchase vaccines at a discount, which are distributed to state health departments and certain local and territorial public health agencies [15, 16]. These agencies then distribute them at no charge to doctors’ practices and public health clinics registered as VFC providers [15]. Fees for administering the vaccines may be covered by Medicaid or private insurance but in some cases are paid out a pocket by parents/caregivers [16].

In Texas, the HPV vaccine is not required for school entry and thus is considered a “recommended” vaccine [14]. HPV vaccination rates in Texas are also lower than most other states, ranking 47th nationwide [12]. Texas Latino/a 13–17 year-olds compared to U.S. Latino/a adolescents had lower HPV vaccine completion (45% vs. 54%) rates [13]. Texas Latino/a 13–17 year-olds compared to U.S. Latino/a adolescents had similarly high rates for required vaccinations (MenACWY 87% vs. 86%; Tdap 85% vs. 87%), which highlight the vaccination disparity [13]. Of note, the HPV vaccine completion rate also falls short of the Healthy People 2020 target of 80% and the Texas Cancer Plan target of 45 to 60% for adolescents ages 13 to 15 completing the series [17–19]. Given the Latina health disparities in cervical cancer incidence and mortality in South Texas, a public health priority is to increase HPV vaccine uptake among Latino/a adolescents [20].

Reviews of the HPV vaccination literature [21, 22] indicate provider recommendation is a strong predictor of HPV vaccine uptake among adolescents. However, there are recognized barriers to provider’s making an effective HPV vaccine recommendation, which involves the provider recommending the HPV vaccination the same way and on the same day as a he/she recommends other vaccines for adolescents [23]. Research involving providers of urban, low-income, and/or mixed race/ethnicity adolescents patients indicate provider-related factors include lack of awareness, understanding, and recommendation of the HPV vaccine [24] and of risks associated with HPV-related diseases among males [25], negative attitudes towards vaccinating males, and anticipated parental resistance [26]. Other provider-centered factors contributing to low HPV vaccine uptake include the fact that the vaccine is only being recommended, rather than being required [26]; the limited time allocated to patient visits [27, 28]; and the fact that most patient visits address acute care concerns [27]. In their literature review of factors associated with HPV vaccine uptake, Jeudin and colleagues [29] found that low-income and ethnic minority adolescents were equally as likely as their white and higher-income peers to begin the vaccine series, but were less likely to complete series and less likely to report that their provider recommended the vaccine to them [29].

Qualitative research on HPV vaccination is limited to examinations of healthcare providers of urban, ethnic minority adolescents with the goal of identifying barriers and facilitators of HPV vaccination and their influence on uptake [30, 31]. Providers interviewed in Javanbakh and colleagues’ [30] study reported parental beliefs and misconceptions (e.g., vaccines not needed for adolescents and concerns it promotes sexual activity) regarding the HPV vaccine as the main barrier. Other barriers included immigrant parents not having their child’s immunization records and high patient mobility and difficulty in reaching the target population [30]. Providers from Katz et al.’s [31] focus groups reported a variety of systems-level challenges ranging from lack of standardizing vaccine administration, time constraints in clinic, and the need to have collaboration between providers. Providers also noted perceived negative beliefs towards the vaccine from caregivers due to concerns about promoting early sexual activity [31].

This study describes HPV vaccination in the context of the socio-cultural and economic realities faced by providers in South Texas using qualitative description (QD). Given the status of Texas’ HPV vaccination rates and lack of literature addressing HPV vaccination among rural Latinos, this research described providers’ perceptions on the process of administering the HPV vaccine to Latino adolescent patients and also identified specific barriers and facilitators they experienced within the context of their clinic practices, which could potentially impact HPV vaccine uptake. We selected QD to address this research gap because it is appropriate for discovering the who, what, and where of experiences and gaining insights from professionals regarding a poorly understood health phenomenon [32–34].

## Methods

The current study was part of a broader research project (NCI; K01 CA181530) focused on developing and testing the feasibility of a theory-based, evidence-informed, culturally relevant intervention to promote Latino adolescent HPV vaccination. We have published elsewhere [35] our findings regarding predictors of Latino mothers’ initiating and completing the vaccine series for their child. To describe the process of how providers administer the HPV vaccine in their practices, we used qualitative description or QD [32–34]. Researchers have used QD to describe the experiences of healthcare professionals and their views on patient-professional interactions and the organization of the health system in relation to particular health phenomena [36] or interventions [37]. The specific aims were (1) to explore healthcare providers’ perceptions of the process of administering the HPV vaccine to patients in their practices within the geographical and cultural context of South Texas; and (2) to identify facilitators and barriers to administering the HPV vaccine to Latino/a adolescents that may shape Latino parent’s perceptions and decisions to vaccinate their child.

### Participants

The University of Texas (UT) Health San Antonio Institutional Review Board approved the study and the research staff obtained informed consent from all participants prior to data collection. The principal investigator (PI) had a partnership with Dr. K., the lead of the medical student clerkship at the UT Health San Antonio Department of Pediatrics. Dr. K. had a network of medical student practicum mentors who either had private practices or worked at Federally Qualified Health Center clinics. Dr. K. emailed his network of medical student practicum mentors (*n* = 29) that they would be receiving a recruitment email from the PI (DMC) informing them about the current study. The PI then emailed the formal invitation to participate in the study and asking participants to email the PI if interested in participating. This initial email recruitment yielded 10 participants. At the end of these interviews, we asked participants to recommend and provide contact information for other providers who would be interested in participating. Subsequently, through this snowball referral method, we recruited five additional participants.

The final sample of 15 participants included ten physicians located along Texas-Mexico border and four nurse practitioners and one physician whose practices were located in Central Texas. The majority of providers were doctors (*n* = 11, 73%), male (*n* = 8, 53%), self-identified as white (*n* = 10, 67%,) and ethnicity as Hispanic (*n* = 8, 53%). Participants ranged in age from 36 to 71 years (median = 49). The majority (*n* = 11; 85%) indicated English as their primary language and reported primarily speaking English with their patients (*n* = 9, 60%). Nearly half identified both their practice type as primary care pediatrics (*n* = 7, 47%) and employment at a Federally Qualified Health Center (*n* = 7, 47%).

### Data collection and analysis

Data collection occurred between January and July 2016. A research assistant scheduled and was physically present at all interviews. During the interview, the research assist collected demographic information (i.e., age, gender, race/ethnicity, language spoken, country of birth, years living in the United States, and their practice), provided a copy of the consent form, managed the audio-recording of each interview, and recorded detailed observational field notes in English. The PI and lead author (DMC) called each participant, obtained verbal informed consent, and personally conducted each of the 15 individual, in-depth, telephone interviews.

The semi-structured interview guide consisted of 28 open-ended questions related to four topics: the provider’s usual clinical practice, HPV vaccine guidelines in the practice, the provider’s personal attitude and perception of the community attitude towards HPV vaccination, and provider communication with patients (Table 1). According to Neergaard et al. [34], the interview guide for qualitative description research is more structured than other qualitative methods and is based on expert knowledge to focus on areas poorly understood in a healthcare context. Interview length ranged from 36 to 73 min, with an average of 56 min. The providers answered all the questions covered in the interview guide. At the conclusion of the interview, each participant signed a voucher to receive $50 check in the mail as compensation for their time, and received published information on HPV vaccination guidelines.

**Table 1:** Sample Questions from Provider Interview Guide

**HPV vaccine guidelines in the practice**	
• Can you describe to me the procedures for administering required vaccines [e.g., Tetanus, Diphtheria, Pertussis (Tdap) and Meningococcal (MCV4)) to preteens (11–12 year olds) and teens (13–18 year olds)] in your practice?	
▪ Follow-up probe: Examples of procedures include ordering and administering vaccines, recall/reminder systems to bring patients in for vaccines, standing orders to improve vaccination, quality improvement procedures to track and increase vaccination rates.	
• How do these procedures compare to the procedures used for the HPV vaccination which is recommended but not required?	
▪ Follow-up probe: For instance, Tdap and MCV4 are required for school entry but HPV isn’t; does this influence how vaccines are ordered or stocked in your practice?	
• What do you know about the HPV vaccine guidelines/recommendations from the Advisory Committee on Immunization Practices or ACIP?	
• Do you think the process of administering the HPV vaccine to patients in practices in your area is ***difficult or easy***?	
▪ Follow-up probe: What factors make it ***easy*** (use the following probes^*^ if needed: financial, legal, behavior change, concerns relative to cancer) to adopt the HPV vaccine guidelines in practices in your area?	
▪ Follow-up probe: What factors make it ***difficult*** (use the following probes^*^ if needed: financial, legal, behavior change, concerns relative to cancer) to adopt the HPV vaccine guidelines in practices in your area?	

^*^ The interviewer only offered probes to the provider when s/he was not able to respond to the question (e.g., staying silent) or asked the interviewer for an example

A professional transcriptionist rendered each audio-recorded interview into a written transcript. The lead author and PI (DMC) uploaded the transcripts into ATLAS.ti 7.0 (Scientific Software Development GmbH, Berlin, Germany), a qualitative software program that facilitate coding and qualitative data analysis. A team of three investigators (DMC, BEF, and SB) conducted the content or thematic analysis by coding and clustering descriptions of the investigative topic within and across transcripts looking for commonalities and differences, while also examining the data in light of existing knowledge [33, 34]. Initially, two team members (BEF and SB) independently coded half of the transcripts, assigning conceptual labels (i.e., codes) to indicate discrete concepts or processes across participants, creating the preliminary codebook. Subsequently each analyst met independently with lead author (DMC) to discuss emerging themes and refine the coding process. These consultations focused on reviewing the coding process, identifying areas of consensus, discussing areas of disagreement, and eventually reaching a consensus regarding each coding category. Subsequently, the lead researcher (DMC) used ATLAS.ti to run summary reports related to HPV vaccine recommendation, provider perception of adoption, provider perception cultural concerns, barriers and facilitators to identify major themes related to frequency among participants. These summary reports show all the quotes coded using a selected code from all transcripts.

After the team had coded and reviewed almost half the transcripts (*n* = 8), it became apparent that there was little variation in the developing themes across the sample, an indication of potential saturation [38]. Each facilitator and barrier was situated on either the parent/patient level or the environmental level. Within the parental/patient level, each theme was identified as either internal or external to the agent and at the environmental level, themes were classified as either policy or organizational. We gave participants pseudonyms and used these in the presentation of quotes.

## Results

Study participants (doctors and nurses) described how they recommended vaccinations to their patients during clinic visits and then had either their nurse or medical assistant administer the vaccine(s) after the visit. A summary of providers’ perceptions of HPV administration facilitators and barriers discussed in this section are presented in Table 2. Key facilitators included parental acceptance/motivation for vaccinating their children, provider motivation of parents, policy (e.g., free, low-cost vaccination and obligatory reporting, and vaccine schedule), and implementing evidence-based practices as facilitators. Other identified barriers included lack of knowledge regarding the HPV vaccine, misinformation from media promoting anti-vaccination, fears related to children engaging in sexual activity, concerns about potential side effects of the vaccine, lack of transportation because of living in remote rural areas, lack of clinic reimbursement for providing the vaccine, and lack of vaccine affordability for patients without insurance coverage.

**Table 2:** Providers’ perceptions of facilitators and barriers to HPV vaccine administration among their patients

	Individual Level (Parent/Patient)	Environmental Level
Facilitators	• Internal-parental personal motivation and acceptance of HPV vaccination	• Policy-free, low cost/obligatory reporting • Policy-given at same time as required vaccines
	• External-parent motivated by provider recommendation	• Organizational-practice reminders/standing orders/appointments
Barriers	• Internal-parental knowledge deficits and media misinformation • Internal-parental concerns of child’s sexual activity • Internal-parental and patient/concerns of vaccine side effects	• Policy-HPV vaccine only a recommended vaccine
	• External-parental and patient/perceived financial concerns • External-parent/lack of transportation	• Organizational-Clinic reimbursement/patient affordability

### Perceptions of parental/patient factors as facilitators

Providers identified both parental motivation and receptivity to provider recommendation as facilitators of HPV immunization uptake. Dr. Cruz noted that in many cases parental buy-in was a given: *“I don’t have any difficulty for them to have the vaccine. I don’t even need to tell…[them] it is important and have it done. They will voluntarily go and have it.*” Several providers had observed that a parent’s family history of cancer or personal experience with HPV or cervical cancer as a motivator for vaccinating their child. For example, Dr. Ellis noted *“[t] here are some families that have cancer that is predominant in their families. Those are the ones that…ask for the HPV vaccine, even before I even bring it up. They’re the ones that are all over it and…complete the three-dose series…*.” Dr. Alonso commented, *“The [parents] that want it, it’s usually because they have a personal or close story about somebody with cervical cancer or something HPV-related. They’re actually very eager and actually request it.”*

Some providers noted having encountered parents who were *“very receptive to recommendations by the healthcare providers”* and readily took into consideration the provider recommendation before deciding to vaccinate. The following examples illustrate how several participants associated the importance of having good parental communication skills with the need to encourage parents to consider HPV vaccination: *“I think it’s more confidence and more listening to them carefully and answering them carefully (Dr. Lewis).”* “*…It’s a matter of talking to them in terms that they can understand…*. *(Dr. Alvaro).” “[I]t depends…how well you explain it, how well you communicate with the parents and any myths that they may have (Nurse Rose).”* Dr. Ellis also used the effective strategy of providing a motivating cancer prevention message: *“What helps me [is] when I mention the prevention of cervical cancer, and tell them it’s the third leading cause of cancer world-wide - leaning towards the cancer prevention really does help convince a family decide to go through with [the] HPV [vaccine]…”.*

### Perceptions of economic and organizational facilitators

The majority of providers reported that the HPV vaccine is available for free or low cost to their patients who qualify for the Texas Vaccines for Children program*.* The state covers the cost of the vaccine for clinics *“because the vaccines are provided by the government and we don’t charge for the vaccines,”* and patients who provide proof they qualify for the program can receive the vaccine free of charge. Nurse Rose noted there might be additional costs to patients: *“This is a federal qualified clinic. We don’t charge, we only charge a minimum amount for the visit ($10). We don’t charge for the immunizations.”* However, she noted barriers related to other potential costs: “*Financial, maybe due to lack of transportation but not for the cost of immunization.”*

Dr. Alonso noted that required guidelines associated with statewide immunization funding was a motivating factor operating at the institutional level: *“…The thing that’s made it easier is more of [what] we’re graded on…so unfortunately, it comes down to money. So, if we’re being graded on it, that we have to implement these guidelines to at least offer the vaccine, if not give it.”* Among the providers interviewed, there was general consensus that both the state vaccination requirements and the availability of funding to cover the cost of vaccines facilitated the implementation of HPV vaccine guidelines within their clinic practices.

Other facilitators to expanding HPV vaccine coverage included state-mandated vaccine requirements for 11 to 12-year-old children. Dr. Morrison described the situation as follows:

*So for sure, I would put at the top of the list the fact that our population follows [the vaccine] requirement almost blindly. I don't know if that's the inherent fear that they have of the people in power. But if a school give[s] them a yellow slip to go to the pediatrician, they will come.”*

Nurse Rose aptly noted, *“…whenever you have immunizations that are required by the school system and they come at the same time, it’s easy for them to just get it [HPV vaccine] at the same time.*

At the organizational level, factors facilitating HPV vaccine uptake included clinic practices ranging from streamlined scheduling to parent-friendly operating hours: *“…[T]hey can come in after school. We’re available till 5:00 for vaccines and we do have even the night clinic available. If they need to, they could even come back in our evening clinic if they wanted to (Dr. Norman).”* Other organizational facilitators included dissemination of patient reminders and standing orders:

*I think that in this particular clinic we have a good scheduling department, so when they leave that initial visit with the first series then they do really call the patients at two months just to make sure that they come in for the visit. We also don’t actually schedule them with a doctor for the completion of the series. They just come in for a nurse visit. They just come in and the nurses will get the immunizations ready and they immunize the child. – Dr. Cascabel*

### Perceived parental/patient barriers

Providers identified parental/patient barriers such as knowledge deficits; exposure to misinformation; existing health beliefs such as fear of their child engaging in sexual activity and concerns about potential side effects of the vaccine; lack of transportation, and cost of vaccination.

#### Parental knowledge deficits

Providers identified a number of parental barriers, ranging from lack of knowledge and understanding of basic information regarding the HPV vaccine, to structural and economic barriers. Limited parental educational levels, preconceived ideas, and exposure to misinformation were factors providers identified as contributing to parental lack of knowledge regarding not only the existence of the clinic and the HPV vaccine itself. Providers acknowledged a generalized lack of awareness and knowledge of the HPV vaccine. An example of these providers’ perceptions of knowledge barriers included Dr. Alonso’s observation: *“Most people just don’t know about the vaccine, or if they have, they have just heard one bad thing about it and don’t really research it or ask more questions about it.”* Nurse Kristoff identified parental educational and cultural factors: *“…I think the lack of education, or either the lack of understanding. I know some of our patients have limited education levels, and they have preconceived ideas or cultural barriers.”* She further reflected on her interactions with parents, noting they often want to have time to make a decision, and the inherent risks of allowing parents to take this time: *“And even in trying to talk to some of them, they want to think about it; and then, of course, if you give them too much time to lapse between the visit and thinking about it, they don’t come back.”*

A few providers associated parental exposure to misinformation from the media (e.g., Spanish-language television, Internet) as a concern. Dr. Morrison stated, *“a lot of misinformation ….From my understanding...[it] is the Spanish media…had a lot of information about this being [related to] the sexual stuff*…” Regarding parents seeking information on the Internet, Dr. Alonso described his experience with resistance from more highly educated parents armed with misinformation:

*…We actually find a bigger resistance to the vaccine in our insured population, because they’re looking more at who-knows-what websites that have said “this vaccine does whatever to you,” so our bigger problem [is] giving the vaccines…to…our educated population. [They] refuse it because they’re spending too much time on the internet.*

#### Parental concerns about sexual activity and negative consequences

Providers also discussed the ways in which parents’ concerns of the HPV vaccination contributing to or facilitating a child’s engagement in sexual activity: “*you are giving them the blessings to go have sex, which is the perception, the fear.”* Nurse Kristoff reflected on the difference between how parents and teens may view the HPV vaccine:

*…[A]s a parent, you don't want to think of your 11-year-old being sexually active. As a mom you think, okay, if you give them this shot, is that in their mind then a rite of passage to, ‘Oh, now I'm free from this sexually transmitted disease, so now it's okay to have sex.’?*

The association of the HPV vaccination with birth control was another parental concern that providers encountered*.* Dr. Alonso stated*, “The ones* [parents] *that absolutely refuse it, there seems to be the misconception that getting the vaccine – they think of it as birth control, so it’s like if they* [adolescents] *get the vaccine, that means they can go and have sex.”* Dr. Avis noted a tendency for some parents to equate women getting cervical cancer as “pay back” for having been being promiscuous as adolescents: *“…because [parents] hear that [HPV vaccine] might make them more promiscuous and they don’t want to protect them…It’s this attitude that…if they end up getting cervical cancer, well, for being promiscuous, they kind of deserve it, which is scary.”* Parents who believed their children were not susceptible to HPV-related diseases also concerned providers: *“…[Some] Moms and Dads feel like their daughters are not sexually active and so they don’t really need to give the vaccine and it can wait. And so, I think just postponing it is a big issue…(Nurse Messias)”* Several providers also identified some parents’ concerns about possible side effects from the HPV vaccine as a potential barrier:

*…I think Hispanics are fearful of the unknown. They're not sure of how their child is going to respond to the vaccine, if the vaccine is harmful to their child. They actually want to wait till their child is 18…to make that decision. That's what I've encountered a lot…some are just stuck on: “My child could be permanently disabled or die from this vaccine.”*

While Dr. Morrison related her experience: “*And it is a painful vaccine according to patients*…*We’ve had a couple of adolescent girls, we’ve had one faint and another one get dizzy*….”

#### Perceived financial and transportation barriers

Financial concerns, whether actual or perceived, also created barriers to parents seeking HPV vaccinations for their adolescent children. Dr. Morrison reported increasing parental concerns regarding paying for vaccine among the insured: *“Yeah, that’s part of parental concerns: ‘How much is it gonna cost?’ And we tell them – well, my understanding is you can no longer get ‘em under Vaccine for Children if they have insurance. So that’s a big one too.”* Given the low socio-economic status of the local community, providers recognized the cost of the vaccine as a deterrent for the local community:

*We have a very low-income community here, and I think the main reason why we have a few of these people coming in here is because of the cost. Once they know the cost, you know, they usually decline [the] vaccination. Not because they don't want to, but because of the cost of the vaccine. (Dr. Luna)*

Dr. Avis noted that very few of her college-age patients have insurance coverage and therefore were not able to afford the vaccine:

*They are very few that have insurance. The majority - once you turn 18, you get bumped off of Medicaid, and so the kids don’t have anything….They’re left in limbo...I would say that it is difficult, because we charge for it. I think I would be able to talk a lot more students into getting it if it was free…I’m pretty sure my vaccination rates would be a hell of a lot better.”*

Limited access to transportation, particularly among those living in more remote locations, was another barrier to completing HPV vaccination. Providers noted that the majority of their Latino/a patients live in “*a remote area, rural area, [and] they depend on transportation.”* Nurse Rose attributed the lack of transportation to the high rate of no-shows for immunization visits among her Latino/a patients: *“….I look at my schedule every single day and I say, okay, I have about five or seven of the immunizations coming in. The trouble that I see is the [lack of] transportation because they only show maybe two or three.”*

### Perceptions of policy level clinic barriers

Providers identified two policy-level barriers to HPV vaccine uptake. The first is that HPV is a recommended, but not required, vaccine. Providers aptly noted that required immunizations are associated with more severe consequences, and that “*when you get to the HPV and the nurse is writing in parentheses “recommended,” then that becomes a whole different issue.*” Dr. Lewis explained how this plays out in his practice:

*Parents understand there are fearful consequences associated with not “following the recommendation that the school is asking them to follow. It's very simple. [The HPV vaccine] is not compulsory. That's it. Nobody understands the vaccine. If it's compulsory, you give it for the school, let's start. If it's not compulsory, I'll think about it and I'll come back, and they don't come back. It's a compulsion by the school. That's what makes the other vaccines perfect.*

Several providers also identified specific insurance-related barriers. These included insurance plans with required patient deductibles for the HPV vaccine, or challenges related to qualifying parents/children for federal programs to cover the vaccination cost. Dr. Morrison noted the challenges even insured patients face:

*They don't make it clear to parents…so sometimes…if they have insurance, we're not able to qualify them for the federal program. So that, I think, is a barrier for insured patients. They're always concerned about, "How is this getting paid? How much am I gonna have to pay?" That type thing.*

Providers also encountered several organizational hassles, ranging from not having the HPV vaccine in stock to problems obtaining state reimbursement for the cost of the vaccines. At the institutional level, vaccine costs were a significant barrier:

*…[I]f we're able to get it in a way that doesn't break the bank for us and insurances are gonna reimburse us and the state provides us without just a huge amount of hassle, then those are the only barriers that I can see… (Dr. Morrison)*

According to Nurse Kristoff, not always having the vaccine in stock was a barrier for her patients, particularly those with transportation issues:

*As long as we have the vaccines available, so no one has to wait for them, you know, be asked to come back, because I know that's a deterrent because we do live in rural Texas out here. So, coming back might mean a 35-minute drive.*

Working at a practice that did not have the HPV on site, Nurse Messias reported all her patients had to be referred to the public health department. She noted both the extra work this created for her and the increased burden on the health department and the patients: *…Generally, I think it’s been left up to the health department to do it. I can’t* [provide the vaccine]*…then,* [to obtain the HPV immunization at] *the health department, you have to call and make an appointment, and then you have to remember to go there and get your vaccine. So, I think it’s pretty difficult.*

## Discussion

These research findings demonstrate the multifaceted nature of both facilitators and barriers regarding the process of HPV vaccine administration and their influence on uptake among Latino/a adolescents in South Texas, factors that healthcare providers encounter during their interactions with patients and their parents, as well as at the organizational and policy levels. Based on their interactions with both parents and adolescents, providers noted that parental beliefs and motivations could serve as facilitators. Examples included having a personal or family history of HPV or cervical cancer serving as a potential motivating force to ensure that a child received the vaccine. Javanbakht and colleagues [30] reported similar findings from their analysis of provider interviews regarding a parent’s family history of abnormal Pap or cervical cancer. They noted providers recognized that parents were receptive to messages that encouraged their consideration of the vaccine. In their analysis of patient-provider interviews, Hudson et al. [39] reported similar approaches to effective communication techniques, including specifically engaging in didactic conversation and demonstrating awareness of cultural and practical barriers to immunization series completion. Our findings suggest that both the information provided and a positive patient-provider rapport could facilitate HPV vaccination compliance. This research highlights the importance of good provider/patient communication skills and the need to promote scripts that may help clinicians to effectively recommend and answer questions regarding the HPV vaccine.

Similar to previous findings related to providers’ attitudes and practices reported by Hudson et al. [39] and Head et al. [40], these South Texas providers identified specific organizational practices and policies, including reminders, standing orders, and pre-booking patient appointments for future doses, as enhancing vaccination efforts. Javanbakht et al.’s [30] findings based on interviews with providers serving a predominantly Latino population, indicated providers perceived parental misperceptions that vaccine programs only covered younger children. In contrast, these South Texas providers reported not only did parents access state-funded programs to cover the costs of HPV vaccination, but also that receipt of these state funds contributed to better accountability among providers in terms of reporting immunizations. Of note, providers did state specific barriers related to their ability to cover the cost of the vaccine for college-age students.

Barriers identified by these research participants ranged from the parent/patient level to the macro-environmental level. South Texas providers described the challenges they faced when parental lack of knowledge or misinformation created difficulties or barriers to HPV immunization uptake. Similarly, Katz et al. [31] reported caregivers lacked education on the HPV vaccine, which contributed to their reluctance to consent to give their child the vaccine. In their analysis of interviews with rural providers in Appalachian Kentucky, Head et al. [40] also found their patients had inadequate vaccine information and did not identify with media promoting the HPV vaccine.

The healthcare providers who participated in this study noted a wide range of parental concerns. These included the belief that providing adolescents with the HPV vaccine could contribute to an increase in the adolescent’s sexual activity. Other observations were that whereas some parents did not perceive their child as being susceptible to HPV-related disease, in contrast, others considered a cervical cancer diagnosis as retribution for their daughter’s “bad” behavior. Morales-Campos and colleagues [41] previously reported that parents tend to believe their child is not at risk for HPV and HPV-related diseases and that parents do not think that HPV is a threat to their daughters’ health. These findings regarding the perception that HPV vaccination contributes to increased sexual activity are similar to those from numerous other studies [30, 31, 40] among diverse groups of providers and parents. A finding of concern was providers’ reporting that some parents may intentionally refuse to vaccinate a daughter as a way to enforce the threat that she “deserves” the punishment of getting cervical cancer as a result of her “promiscuous” behavior. Clearly, further investigation of such beliefs and attitudes is warranted in order to better understand how the potential negative impact of stigmatization on public health efforts.

These findings indicated common barriers to effective HPV uptake include actual or perceived side effects, declining or delaying vaccination, perceived financial barriers, and lack of transportation. Of note, these findings echo results from other prior qualitative research. For example, Head et al. [40] reported fear of vaccine-related pain as a potential deterrent to HPV uptake and Morales-Campos and colleagues [42] documented similar concerns among Latino parents about vaccine safety and side effects. Findings from prior research on providers’ vaccination concerns include the lack of parental education about vaccine efficacy and safety [31]. Javanbakht and colleagues [30] reported providers attributed their patients’ vaccination concerns to the newness of the HPV vaccine, which resulted in their deciding to delay having their children vaccinated. Similarly, these findings indicated some parents preferred to defer HPV vaccination for their children until age 18, at which age they were deemed old enough to make the decision for themselves. Given that the HPV vaccine guidelines recommend vaccination at the age of 11 or 12 years, before the onset of sexual activity, such attitudes and behaviors are concerning. Furthermore, providers perceived waiting to vaccinate older adolescents as problematic because these adolescents may choose not to receive it or delay it. In this respect, our findings contradict Perkins et al.’s [25] report that providers reported conservative parents of girls as tending to defer HPV vaccination because of its association with sex, in contrast to boys’ parents, who did not defer HPV vaccination.

Not surprisingly, providers who participated in this research perceive parents to be unaware of available support to cover the costs of immunizations. Our findings indicated providers tended to perceived parents and patients as being unaware of the availability of programs that cover the cost of vaccinations for both children and young adults. Echoing these perceived financial barriers, Javanbakht and colleagues [30] reported that parents believed Vaccines for Children and other similar initiatives were only available for younger children. Lack of transportation was an acknowledged barrier to accessing and completing the HPV vaccine series among Latino families living in South Texas, a finding similar to other previous research on cervical cancer prevention programs among Latinos living along the Texas-Mexico border [35, 43].

There is a clear need for ongoing research conducted in clinic settings to understand the communication dynamics between adolescent patients, their parents, and providers. In order to effectively inform and enhance public health funding for HPV prevention and control efforts, it is necessary that future research initiatives address the multiple barriers and contribute to enhancing identified facilitators in order to determine which combination of factors explain the greatest variance in both vaccine resistance and uptake.

There are several limitations related to both the research design and findings. The convenience sample of participants consisted of a relatively small number of providers who provided medical services to a predominantly Latino/a rural adolescent population in South Texas. All the providers interviewed volunteered to participate. It is possible that given the self-selection process, providers with negative attitudes towards the aim or appropriateness of HPV vaccination may have purposefully refrained from participating. The findings reflect only providers’ perspectives on facilitators and barriers to HPV vaccination, and do not include the views of other key constituents, including adolescent patients and their parents or guardians. Finally, the findings must be interpreted within the constantly changing healthcare environment, especially given the recent COVID-19 pandemic, which initially caused decreases in the number of patient visits to practices (J. Garcia, personal communication, August 30, 2020). Despite these limitations, these findings offer a unique view into the contexts in which providers serve this group of Latino/a adolescents and their parents.

## Conclusions

This qualitative study enhances the understanding of local clinicians’ perceptions of opportunities and challenges for administering the HPV vaccine to their Latino/a adolescent patients and contributes to the current body of literature regarding the effective implementation of available resources to effectively increase HPV vaccine uptake among vulnerable groups and communities. The findings indicate that the key barriers and facilitators to HPV vaccine uptake in South Texas are similar to those previously identified in the literature (e.g., effective patient-provider communication is crucial) but with unique features (e.g., an awareness among South Texas parents that state-funded programs would pay for the vaccine). Furthermore, these results confirm the need for ongoing efforts to continually enhance tailored communications directed towards parents and patients in order to improve public understanding of and confidence in the HPV vaccine. Beyond confirming findings of several previous studies, this research reiterates the importance of messaging that reassures parents that HPV infection should not be equated with a moral punishment. This research clearly supports the need for broader policy changes aimed at addressing the various organizational and structural challenges to implementing and sustaining effective HPV vaccine coverage. Although simple administrative strategies such as pre-booking appointments are effective, even the most streamlined appointment procedures and optimal patient-provider relationships cannot facilitate uptake in the absence of available vaccines.

## Data Availability

The transcripts that support the study conclusions are unavailable for public access because informed consent to share complete transcripts (beyond the research team) was not obtained from study participants, but the de-identified data is available from the corresponding author on reasonable request.
